# AN EDUCATIONAL PROGRAM IN ASSISTED LIVING TO INCREASE STAFF SELF-EFFICACY IN PROVISION OF DEMENTIA PALLIATIVE CARE

**DOI:** 10.1093/geroni/igad104.1122

**Published:** 2023-12-21

**Authors:** Debra Dobbs, Hongdao Meng, Jessica Yauk, Harleah Buck, William Haley

**Affiliations:** University of South Florida, Tampa, Florida, United States; University of South Florida, Tampa, Florida, United States; University of South Florida, Tampa, Florida, United States; University of Iowa, Iowa City, Iowa, United States; University of South Florida, Tampa, Florida, United States

## Abstract

Advance care planning among persons with dementia, their family members and staff in assisted living (AL) communities can facilitate meeting care preferences. In a recent NIH Stage 1a feasibility cluster randomized trial in AL communities (N=118 residents with dementia, 23 staff; k=10 ALs). The Palliative Care Education in Assisted Living for Dementia Care Providers (PCEAL-DCP) intervention showed an increase in documentation of ACP discussions (e.g., completion of advance directives, having goals of care discussions with family) in the treatment group from 12% to 51% compared to control group (8% to 23%) from baseline to six-months (effect size 0.86, Cohen’s d statistic). The current study is an NIH Stage 1b pilot using the same sample to test staff self-efficacy as a mechanism and if staff self-efficacy mediates the effects of increased documentation of ACP discussions. The measure of staff self-efficacy includes two self-reported survey items at pre- and post-intervention (baseline and one-month) that asks them how much they agree with their ability to provide care for terminally ill persons and terminally ill persons with dementia (completely agree, somewhat agree, completely disagree). Descriptive results indicate a larger increase for the treatment AL staff from baseline to one month for the percentage who completely agreed with both items. For the item specific to providing care for terminally ill persons with dementia, the treatment group was 47% baseline to 84.6% one-month compared to 66% baseline and 50% control group. Results for mediation between staff-self efficacy and ACP will be discussed.

